# Strain-Transcending Inhibitory Antibodies against Homologous and Heterologous Strains of Duffy Binding Protein region II

**DOI:** 10.1371/journal.pone.0154577

**Published:** 2016-05-04

**Authors:** Sudarat Wongkidakarn, Amy M. McHenry, Jetsumon Sattabongkot, John H Adams, Patchanee Chootong

**Affiliations:** 1 Department of Clinical Microbiology and Applied Technology, Faculty of Medical Technology, Mahidol University, Bangkok, Thailand; 2 Department of Biology, Southwestern Adventist University, Keene, Texas, United States of America; 3 Mahidol Vivax Research Unit, Faculty of Tropical Medicine, Mahidol University, Bangkok, Thailand; 4 Department of Global Health, University of South Florida, Tampa, Florida, United States of America; Agency for Science, Technology and Research—Singapore Immunology Network, SINGAPORE

## Abstract

Duffy binding protein region II (DBPII) is a promising vaccine candidate against vivax malaria. However, polymorphisms of DBPII are the major obstacle to designing a successful vaccine. Here, we examined whether anti-DBPII antibodies from individual *P*. *vivax* exposures provide strain-transcending immunity and whether their presence is associated with DBPII haplotypes found in patients with acute *P*. *vivax*. The ability of antibodies to inhibit DBL-TH-erythrocyte binding was tested by COS7 erythrocyte binding inhibition assay. Seven samples of high responders (HR) were identified from screening anti-DBPII levels. HR no.3 and HR no.6 highly inhibited all DBL-TH binding to erythrocytes, by >80%. Antibodies from these two patients’ plasma had the potential to be broadly inhibitory against DBL-TH1, -TH2, -TH6, -TH7, -TH8 and -TH9 haplotypes when plasma was serially diluted from 1:500 to 1:2000. To further examine the association of DBPII haplotypes and the ability of antibodies to broadly inhibit DBL-TH variants, the individual samples underwent sequencing analysis and the inhibitory function of the anti-DBPII antibodies was tested. The patterns of DBPII polymorphisms in acute patients were classified into two groups, DBPII Sal I (55%) and DBL-TH variants (45%). Plasma from Sal I and DBPII-TH patients who had the highest inhibition against Sal I or DBL-TH4 and -TH5 was serially diluted from 1:500 to 1:2000 and their inhibitory capacity was tested against a panel of DBL-TH haplotypes. Results provided evidence of both strain-transcending inhibition as well as strain-specific inhibition by antibodies that blocked erythrocyte binding against some DBL-TH variants and against homologous alleles. This study demonstrated broad inhibition by anti-DBPII antibodies against DBL-TH haplotypes in natural *P*. *vivax* exposed individuals. The identification of conserved epitopes among DBL-TH may have implications for vaccine development of a DBPII-based vaccine against diverse *P*. *vivax* infections.

## Introduction

The malaria asexual cycle involves repeated cycles of parasite growth and subsequent destruction of host erythrocytes. Each cycle is characterized by production of merozoites which recognize and invade new erythrocytes for continuation of the parasite cycle. The clinical manifestations of malaria are associated with asexual erythrocytic stages of the parasites [1]. Therefore, targeting these stages may help reduce clinical symptoms during malaria. The merozoite proteins, which play a role in parasite invasion, are important candidates for vaccine development to block parasite invasion and limit blood- stage growth.

The *Plasmodium vivax* Duffy Binding Protein (PvDBP) is released from micronemes during initial attachment of merozoites to the erythrocytes and for junction formation to complete the invasion process. The critical binding motif of PvDBP is referred to as DBP region II (DBPII) or the DBL domain. The interaction between DBPII and its cognate receptor, the Duffy antigen/receptor for chemokine (DARC) on the reticulocyte surface is required for parasite invasion [2, 3]. Naturally acquired anti-DBP antibody are widespread in people living in malaria endemic area and these antibodies can block DBP-erythrocyte binding and inhibit parasite invasion in short-term culture [4–7]. These data support the potential of DBP as a key vaccine development against blood-stage *P*. *vivax* malaria. However, analysis of genetic diversity of *dbpII* alleles among *P*. *vivax* isolates from different geographical regions showed high rates of polymorphisms. Therefore, it may hamper vaccine development as some variant residues alter immune recognition of the DBP antigen.

DBPII polymorphisms significantly change antigenic character and sensitivity to neutralizing antibodies [8, 9]. It has been shown that dominant B-cell epitopes in DBPII are polymorphic surface exposed motifs. These dominant polymorphic epitopes tend to create an inherent bias toward a strain specific immune response [6, 10]. However, this parasite immune evasion mechanism can be overcome since some individuals exposed to *P*. *vivax* in endemic areas are capable of producing broadly inhibitory anti-DBPII antibodies [5, 11]. Therefore, an alternative approach to design DBPII vaccine is to focus the immune response toward conserved epitopes targeting strain-transcending immunity. A recent study indicated cross immunity of anti-DBPII neutralizing antibodies. The DEKnull vaccine candidate had the potential to induce broadly neutralizing antibodies capable of inhibiting heterologous *dbpII* alleles. The removal of dominant polymorphic DEK variant epitopes tended to focus development of immune responses towards the more conserved neutralizing epitopes in the native Sal I strain [12]. Therefore, DBPII based immunogens that target the immune response to conserved functional epitopes may be necessary to avoid induction of strain-specific responses to dominant variant epitopes.

To approach DBPII vaccine development in Thailand, serological responses and inhibitory function of anti-DBPII antibodies were characterized in natural *P*. *vivax* exposures. Individuals produced anti-DBPII antibodies that significantly increased in titer during acute infection but there was no correlation between antibody titer and inhibitory function [13]. Polymorphic patterns of Thai isolates were defined into 9 haplotypes (DBL-TH1, -TH2, -TH3 …etc). The polymorphisms of DBL-TH variants changed the specificity of naturally acquired antibodies. A monoclonal antibody against the DBP7.18 variant (accession no. AAL79051.1) inhibited heterologous DBL-TH haplotypes, indicating anti-DBPII antibodies recognize conserved epitopes that are shared between DBPII Thai variants [14]. Therefore, optimization of immunological responses to conserved DBL-TH epitopes in order to induce broadly inhibitory anti-PvDBPII neutralizing antibody is necessary for effective vaccine development against diverse *P*. *vivax* in Thai endemic areas. The present study was designed to evaluate whether sequence polymorphisms in *dbpII* genes among *P*. *vivax* Thai isolates affect the recognition and specificity of naturally occurring antibody against DBPII Thai variant antigens. An association between DBPII polymorphisms and anti-DBPII inhibitory response was observed in acute *P*. *vivax* patients infected with Thai isolates.

## Results

### Inhibition activity of anti-DBPII antibodies against DBL-TH binding

**Fig 1:**
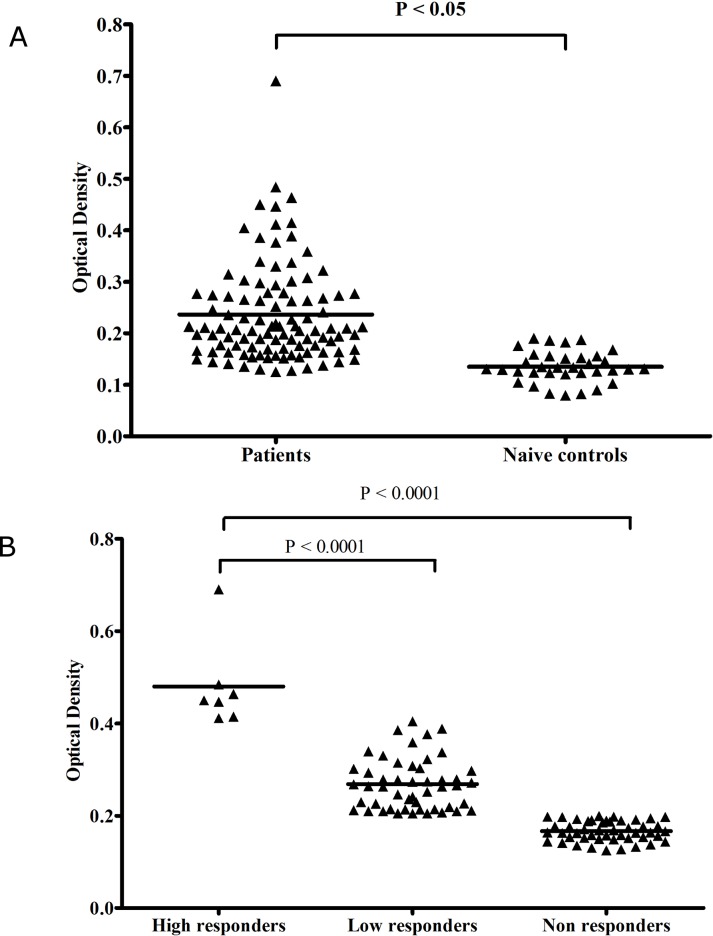
Antibody recognition of recombinant PvDBPII. The scatter plot graph shows the anti-DBPII antibody levels in Thai patients compare to naive control as measured by ELISA. **(A)** Anti-DBPII levels were significantly higher in patients with acute *P*. *vivax* than in naive controls, **(B)** ELISA data classified patients into 3 groups: high responder (HR), low responders (LR) and non-responders (NR). Each dot represents the mean of optical density values in double wells for each sample. The line represents the mean value. Significance was determined by non-parametric analysis using the Mann-Whitney U test. The level of significant was set at *P* < 0.05.

To evaluate inhibitory response against DBL-TH haplotypes in high anti-DBPII responders, the reactivity of naturally acquired antibodies in vivax patients (n = 103) was tested against recombinant DBPII protein by ELISA. The anti-DBPII antibody levels in acute human plasma were significantly higher than naïve controls (*P*. *vivax* patient, overage optical density [OD] = 0.25 ± 0.08, naive controls, OD = 0.13 ± 0.030, *P* < 0.05, Fig 1A). The serological responses to DBP were used to classify patients into three groups; high responders (HR) (OD = 0.41 to 0.69), low responders (LR) (OD = 0.20 to 0.40) and non-responders (NR) (OD < 0.20) (Fig 1A and 1B). The samples were considered positive when OD value was greater than or equal to the mean plus 2 standard deviations of naive controls. There were 7, 49 and 47 patients in the high responder, low responder and non-responder categories, respectively (Fig 1B).

**Fig 2:**
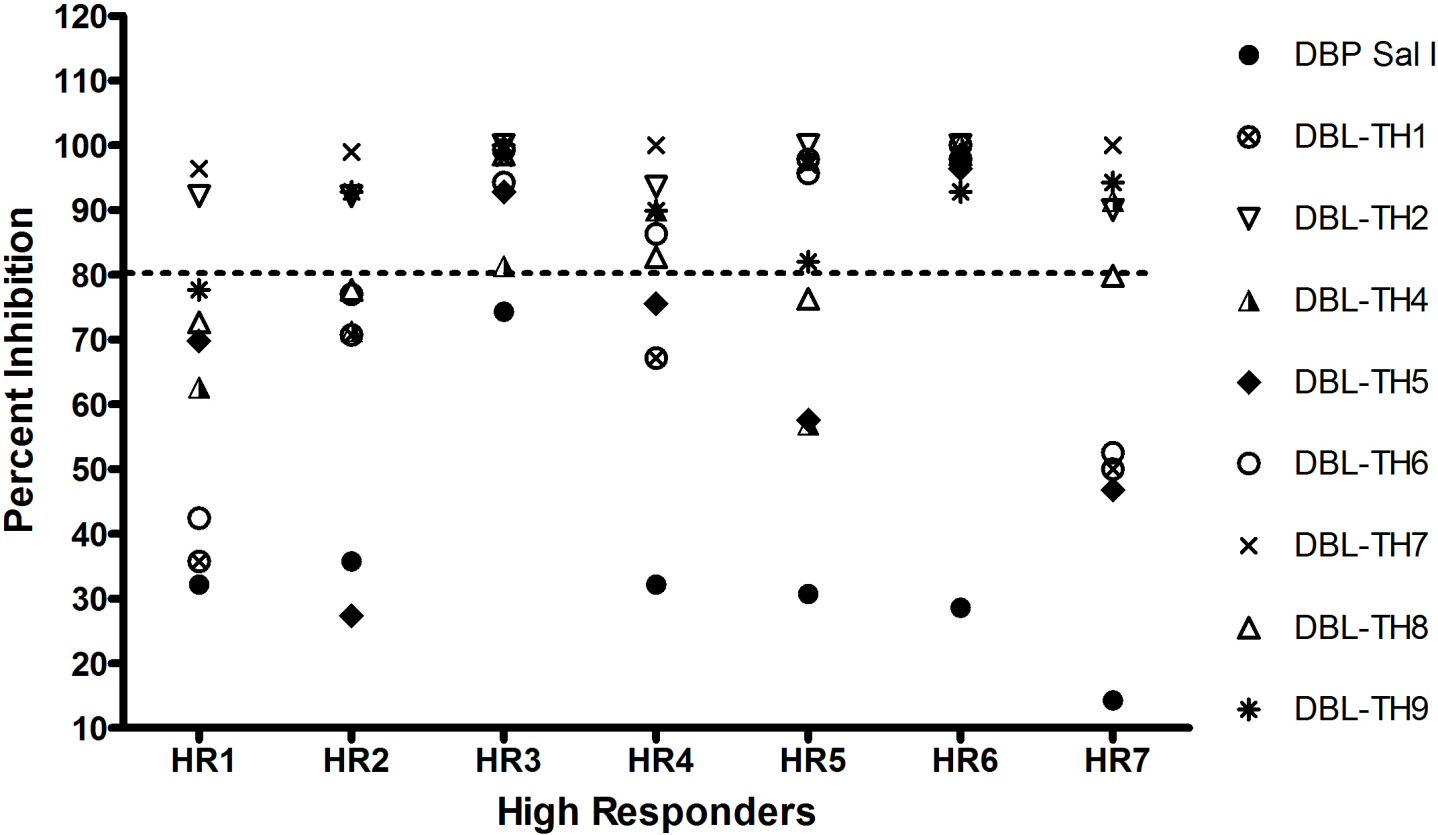
Functional inhibition of anti-DBPII antibodies in high responder samples against the panel of DBL-TH variants. Transfected COS7 cells expressing DBL-TH alleles, DBL-TH1, -TH2, -TH3, -TH4, -TH5, -TH6, -TH7, -TH8, -TH9 and reference Sal I were incubated with 1:100 plasma dilution for 1 hr at 37°C followed by incubation with a 10% suspension of human erythrocytes for 2 hrs. The number of rosettes was compared between wells of transfected cells incubated with plasma relative to wells without plasma (30 fields of view, magnification ×200). The symbols represent mean percent inhibition of two experiments tested in duplicate wells.

Our previous study identified the common DBL-TH haplotypes among *P*. *vivax* isolates [14]. Therefore, in this study, the individual plasma samples of high responders (HR) (n = 7) were used to evaluate inhibitory function of neutralizing antibody against a panel of DBL-TH haplotypes by COS7 cell binding-inhibition assay. All HR patients strongly inhibited DBL-TH2 and -TH7 binding to human erythrocytes, >80% inhibition activity (Fig 2). Most high responders had no inhibitory activity against DBL-TH5 variant binding with only HR3 and HR6 inhibiting this variant. There was no HR patient that inhibited reference DBPII Sal I binding to erythrocyte at >80%. Interestingly, two high responders, HR3 and HR6, inhibited binding of all DBL-TH variants by >80% inhibition activity (Fig 2).

### Broad inhibition of anti-DBPII antibodies against DBL-TH haplotypes

**Fig 3:**
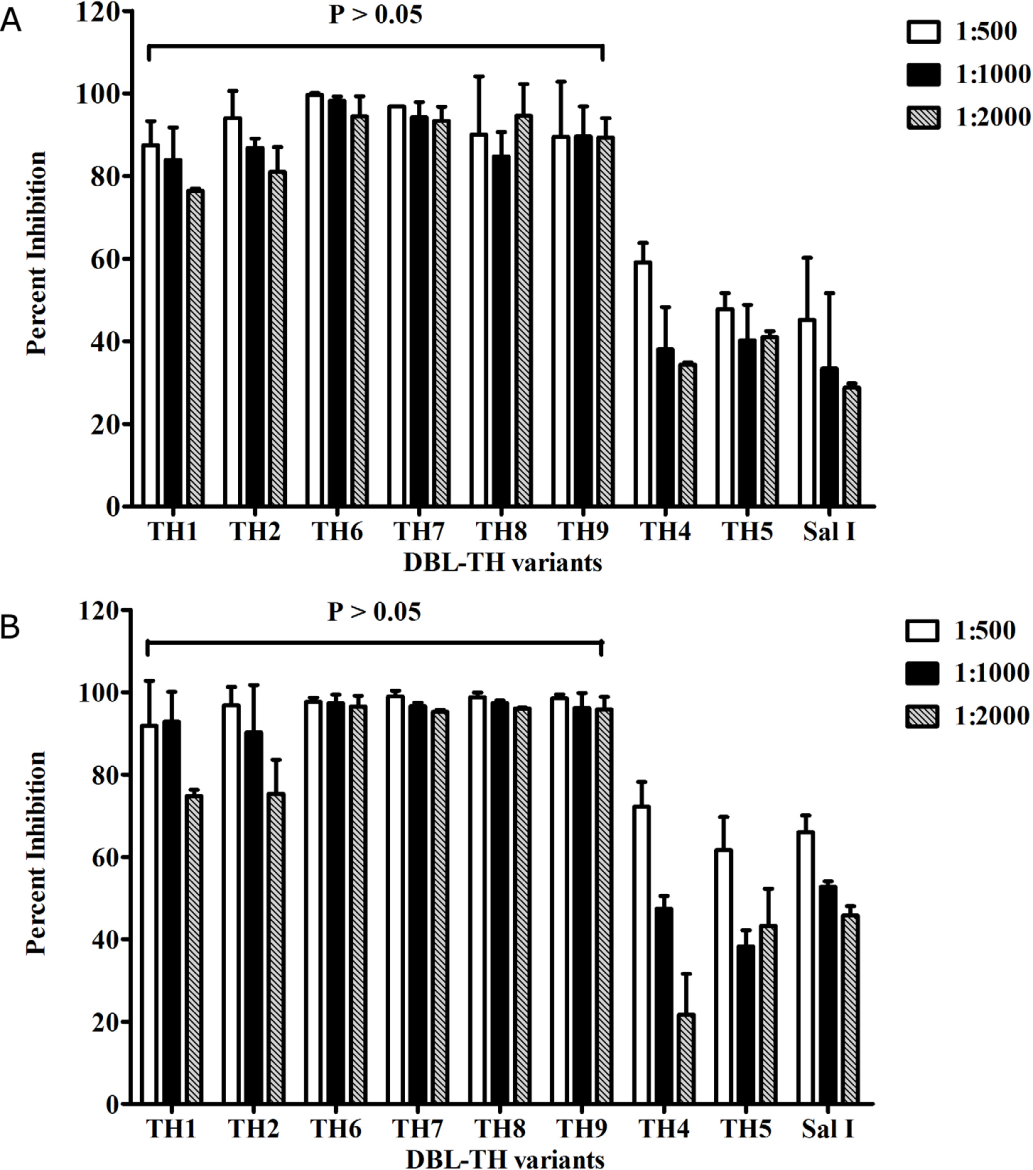
Broad inhibition by high responder samples of erythrocyte binding to DBL-TH haplotypes. The transfected COS7 cell expressing DBPII reference Sal I or DBL-TH variants were pre-incubated with plasma at dilution 1:500, 1:1000 and 1:2000 for inhibition of DBPII-erythrocyte binding. The charts show the mean inhibition of each DBL-TH variant. Inhibitory function against the panel of DBL-TH variants by (A) HR3 and (B) HR6 samples. Each chart represents the mean of two independent experiments with each dilution tested in triplicate. Error bars represent ± standard deviation. Statistical significance was determined using one-way analysis of variance (ANOVA) and multiple comparison analysis by Bonferroni test.

To further analyze the ability of plasma samples from HR3 and HR6 (Fig 2) to inhibit both homologous and heterologous DBL-TH haplotypes, plasma from HR3 and HR6 were serially diluted from 1:500 to 1:2000 and tested for inhibitory function of anti-DBPII antibodies by COS7 cell binding-inhibition assay. The result showed that antibodies in HR3 (Fig 3A) and HR6 (Fig 3B) strongly inhibited DBL-TH1, -TH2, -TH6, -TH7, -TH8, and -TH9, with >80% inhibition at 1:500, 1:1000 and 1:2000 plasma dilutions whereas only low inhibitory efficiency was shown towards DBL-TH4 and -TH5 haplotypes (at 1:500 dilution, HR3; -TH4 = 59.10%, -TH5 = 47.74%, HR6; -TH4 = 72.23%, -TH5 = 61.68%) (Fig 3A and 3B). These data suggest that DBL-TH4 and -TH5 variants are composed of critical polymorphic residues which may change antigenic character and alter the potency of neutralizing antibody. The cross inhibition of antibody against DBL-TH1, -TH2, -TH6, -TH7, -TH8, and -TH9 may indicate the sharing of conserved epitopes among DBL-TH variants.

### The association between DBPII polymorphisms and inhibition of anti-DBPII antibodies

To further evaluate the association of DBPII polymorphisms and inhibition activity of anti-DBPII antibodies in *P*. *vivax* exposed individuals, 40 additional blood samples from acute *P*. *vivax* patients were collected in 2014. DBPII polymorphism analysis was used to identify DBL-TH haplotypes in acute *P*. *vivax* patients and their plasma was used for evaluation of inhibitory activity against DBL-TH haplotypes by COS7 cell binding-inhibition assay. The patterns of DBPII polymorphisms in individuals of acutely *P*. *vivax* patients were classified into 2 groups, those infected with Sal I strain and those infected with DBL-TH haplotypes. There were 22 (55%) and 18 (45%) patients infected with DBP Sal I strain and DBL-TH haplotypes, respectively. For patients infected with DBL-TH haplotypes, 12 haplotypes were defined among *P*. *vivax* isolates. Nine haplotypes (DBL-TH1, -TH2, -TH3, TH4, -TH6, -TH7, -TH8 and -TH9) were similar to our previously reported haplotypes [14]. Three new DBL-TH haplotypes, DBL-TH10, DBL-TH11 and DBL-TH12 were first identified in this Thai endemic area. The highest frequency was DBL-TH1 haplotype (33.33%) (Text in S1 Table).

Since the mutation of amino acids has been shown to change antigenic character of DBPII [8], in this study, DBL-TH4 and DBL-TH5 haplotypes which contain multiple polymorphic residues and reference Sal I strain were used for characterization of broadly anti-DBPII neutralizing antibodies. The result showed that nine (22.50%) patients developed anti-DBPII neutralizing antibody against heterologous DBL-TH4 and -TH5 binding to erythrocytes, >80% inhibition activity. Interestingly, one (2.5%) patients broadly inhibited both heterologous DBL-TH4, DBL-TH5 and holomologous reference Sal I strain (Table 1). In contrast, twelve (30.00%) patients had inhibitory antibody against only heterologous DBL-TH4 or DBL-TH5 strain. One patient (2.5%) developed anti-DBPII neutralizing antibody response against only homologous reference Sal I strain whereas no inhibitory response to heterologous DBL-TH4 or DBL-TH5 (Table 2). Seventeen patients (42.50%) did not develop antibody against DBL-TH4 or TH5 or Sal I binding.

**Table 1:** Inhibitory function of anti-DBPII antibodies in *P*. *vivax* individuals against heterologous DBL-TH4 and DBL-TH5 or heterologous DBL-TH4, DBL-TH5 and homologous reference Sal I binding to human erythrocytes measured by COS7 cell binding-inhibition assay

Sample ID	DBPII haplotypes	Functional Assay		
		DBL-TH4	DBL-TH5	Reference Sal I
2	DBP-Sal I	HI*	HI	NI*
3	DBP-Sal I	HI	HI	NI
9	DBP-Sal I	HI	HI	HI
15	DBP-Sal I	HI	HI	NI
16	DBL-TH1	HI	HI	NI
24	DBP-Sal I	HI	HI	NI
29	DBP-Sal I	HI	HI	NI
31	DBP-Sal I	HI	HI	NI
33	DBL-TH1	HI	HI	NI
36	DBP-Sal I	NI	HI	HI

* NI = Non inhibition (<80% inhibition), HI = High inhibition (>80% inhibition)

**Table 2:** Inhibitory function of anti-DBPII antibodies in *P*. *vivax* individuals against heterologous DBL-TH4 or DBL-TH5 or homologous reference Sal I binding to human erythrocytes measured by COS7 cell binding-inhibition assay

Sample ID	DBPII haplotypes	Functional Assay		
		DBL-TH4	DBL-TH5	Reference Sal I
5	DBL-TH3	NI*	HI*	NI
11	DBP-Sal I	HI	NI	NI
17	DBL-TH11	HI	NI	NI
18	DBP-Sal I	HI	NI	NI
19	DBP-Sal I	HI	NI	NI
21	DBL-TH2	NI	HI	NI
22	DBP-Sal I	NI	HI	NI
25	DBL-TH2	HI	NI	NI
28	DBL-TH8	HI	NI	NI
30	DBL-TH12	NI	HI	NI
32	DBP-Sal I	HI	NI	NI
35	DBP-Sal I	NI	NI	HI
37	DBL-TH4	HI	NI	NI

* NI = Non inhibition (<80% inhibition), HI = High inhibition (>80% inhibition)

### Anti-DBPII antibodies block both homologous and heterologous DBL-TH haplotypes binding to erythrocytes

To support the ability of anti-DBPII antibodies in inhibitory response against both homologous and heterologous during *P*. *vivax* infection, plasma samples from acute *P*.*vivax* patients no.9 and no.15 who had the highest broadly inhibition activity against heterologous DBL-TH4, DBL-TH5 and reference Sal I binding were used to evaluate inhibition against a panel of DBL-TH haplotypes by COS7 cell binding-inhibition assay after serial dilution from 1:500 to 1:2000.

**Fig 4:**
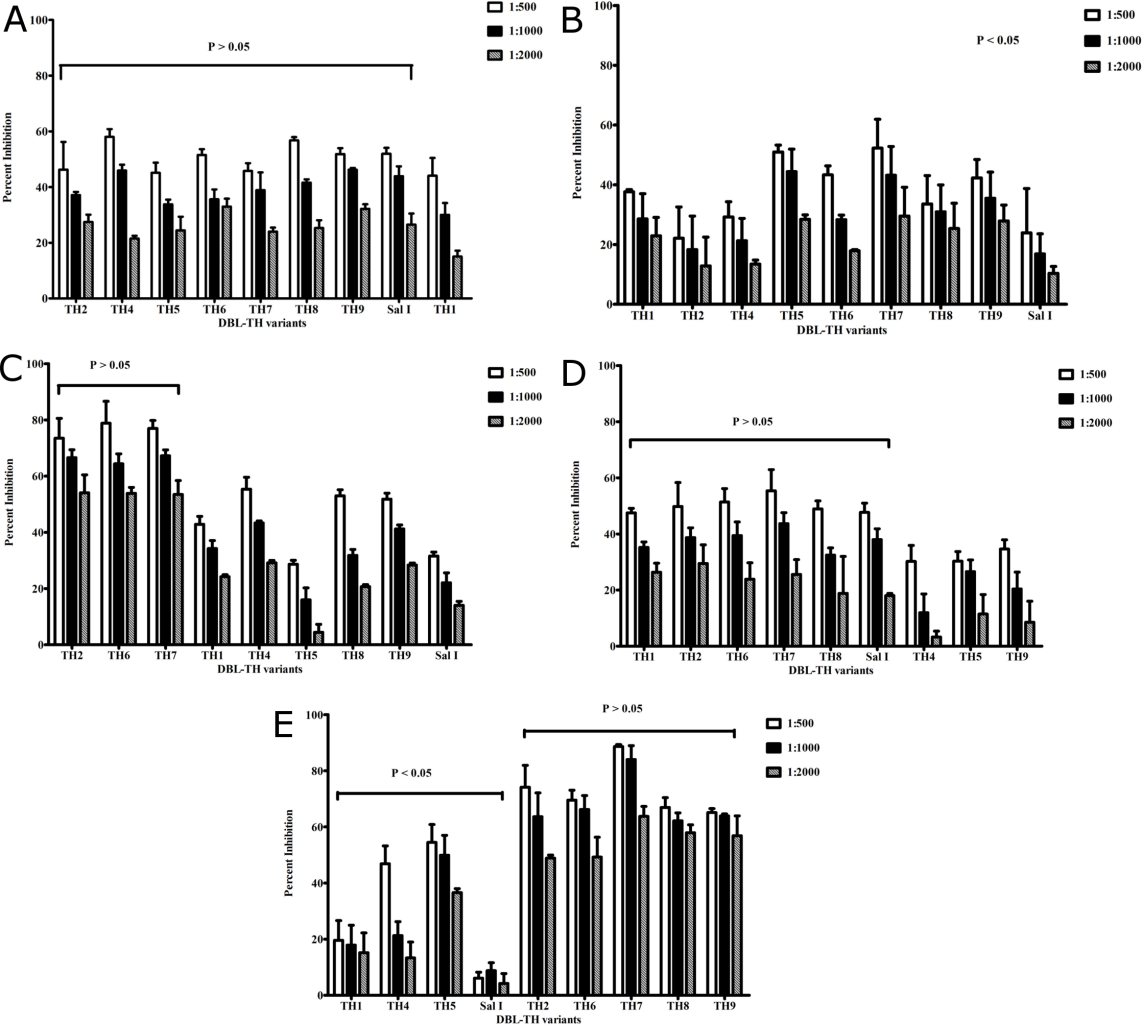
Inhibition efficiency of anti-DBPII antibodies in *P*. *vivax* exposed individuals. A and B: Inhibitory function against a panel DBL-TH haplotypes of vivax patients infected with Sal I strain and had the strongest inhibitory immunity against both heterologous DBL-TH4 and DBL-TH5 strain **(A)** patient no.9, **(B)** patient no.15. C, D and E:. Inhibitory function against a panel DBL-TH of vivax patients infected with high polymorphism DBL-TH strain and had the strongest inhibitory immunity against heterologous DBL-TH4 or DBL-TH5 or Sal I strain, **(C)** patient no.25 infected with DBL-TH2 strain, **(D)** patient no.37 infected with DBL-TH4 strainand **(E)** infected with new DBL-TH strain, patient no.30. The transfected COS7 cells expressing Thai DBPII alleles were incubated with plasma and with human erythrocytes. The number of rosettes was compared between wells of transfected cells incubated with antibodies relative to wells without antibodies. Each chart represents the mean of two independent experiments with each dilution tested in triplicate. Error bars represent ± standard deviation. Statistical significance was determined using one-way analysis of variance (ANOVA) and multiple comparison analysis by Bonferroni test.

For patient no.9, the inhibition capacity typified strain-transcending inhibition showing broad inhibition against heterologous DBL-TH2, -TH4, -TH5, -TH6, -TH7, -TH8, -TH9 and homologous Sal I strain (Fig 4A). Percent inhibition was not significantly different among these DBL-TH haplotypes at 1:500, 1:1000 and 1:2000 dilution (Fig 4A, *P* > 0.05). However, statistical analysis demonstrated significantly different inhibitory activity against DBL-TH1 when plasma was diluted at 1:1000 or 1:2000 (Fig 4A, *P* < 0.05). In contrast, inhibition activity against DBL-TH binding of patient no.15 was not strain-transcending. There was significantly different inhibition against 8 DBL-TH haplotypes and the Sal I haplotype binding to human erythrocyte when plasma was serially diluted from 1:500 to 1:2000 (Fig 4B, *P* < 0.05).

To study broadly inhibition activity of anti-DBPII antibodies in patients who infected with high polymorphism DBL-TH variant strain, patient plasma no.25 who infected with DBL-TH2 and patient plasma no.37 who infected with DBL-TH4 were diluted and tested inhibition capacity against a panel DBL-TH binding. The result showed that patient no.25 who infected with DBL-TH2, showed high inhibition against homologous DBL-TH2 and heterologous DBL -TH6 and -TH7 haplotypes at 1:500, 1:1000 and 1:2000 plasma dilutions (Fig 4C, *P* > 0.05). However, this patient had a low inhibitory immune response to heterologous DBL-TH1, -TH4, -TH5, TH-8, -TH9 and Sal I strain at 1:500, 1:1000 and 1:2000 plasma dilutions (Fig 4C). For patient no.37 who was infected with DBL-TH4 showed broad inhibition against heterologous DBL-TH1, -TH2, -TH6, -TH7, -TH8 and Sal I strain when plasma was diluted at 1:500, 1:1000 and 1:2000 (Fig 4D, *P* > 0.05) whereas inhibition activity against homologous DBL-TH4 and heterologous -TH5, -TH9-erythrocyte binding was very low at 1:500, 1:1000 and 1:2000 plasma dilutions (Fig 4D).

In this study, three novel DBPII Thai haplotypes, DBL-TH10, -TH11 and -TH12 were identified (Table 3). To analyze the association of DBPII Thai polymorphisms and broad inhibition by neutralizing antibodies, plasma from patient no.30, infected with DBL-TH12 haplotype, was examined. Anti-DBPII antibodies in this patient highly inhibited heterologous DBL-TH2, -TH6, -TH7, -TH8 and -TH9 haplotypes at 1:500, 1:1000 and 1:2000 plasma dilutions (Fig 4E, *P* > 0.05). However, the inhibition activity against heterologous DBL-TH1, -TH4, -TH5 and Sal I was significantly different among DBL-TH haplotypes at 1:500, 1:1000 and 1:2000. These data suggest that patients infected with the new DBL-TH variant produced neutralizing antibody specific to conserved epitopes of DBL-TH2, -TH6, -TH7, -TH8 and -TH9 (Fig 4E, *P* < 0.05), but strain-specific antibodies towards DBL-TH1, -TH4, -TH5 and Sal I (Fig 4E).

**Table 3:** Polymorphic residues of DBL-TH haplotypes infected in *P*. *vivax* patients at the time of enrollment.

DBPII alleles	Amino acid position														
	308	313	333	371	375	384	385	386	390	417	424	433	437	475	503
DBPII-Sal I	R	.	L	K	.	D	E	K	R	N	L	.	W	P	I
DBL-TH1	.	.	F	.	.	.	.	.	.	.	I	.	R	A	K
DBL-TH2	.	.	F	E	.	G	K	Q	.	K	I	.	R	.	.
DBL-TH3	.	.	F	.	.	G	.	.	H	.	.	.	.	.	K
DBL-TH4	.	.	F	.	.	G	K	Q	H	K	I	.	R	.	K
DBL-TH5	.	.	.	E	.	G	.	N	.	K	I	.	R	.	K
DBL-TH6	.	.	.	.	.	G	.	H	.	.	.	.	.	.	.
DBL-TH7	.	.	.	.	.	G	.	.	.	.	.	.	.	.	.
DBL-TH8	.	.	F	.	.	.	.	.	.	.	.	.	.	.	.
DBL-TH9	.	.	F	.	.	.	.	.	.	.	I	.	R	.	K
DBL-TH10	.	P	.	.	.	.	.	.	.	.	.	H	.	.	.
DBL-TH11	.	.	F	.	.	.	K	.	.	.	.	.	.	.	.
DBL-TH12	S	.	F	.	D	G	K	N	H	K	I	.	R	.	.

## Discussion

Developing a vaccine for *P*. *vivax* represents a major challenge especially considering the limitation of *in vitro* cultures. To date, a completely effective vaccine that can be introduced to clinical practice for malaria is not available. Most vivax vaccine candidate antigens such as DBP, Merozoite Surface Protein 1 (MSP1) and Apical Membrane Antigen 1 (AMA-1) are highly polymorphic surface proteins that stimulated strain-specific immunity [6, 8, 15, 16]. This antigenic diversity is one of the biggest challenges in vaccine development since it enables the parasite to evade the host immune responses [17, 18]. Therefore, to design an efficient anti-malarial vaccine, worldwide information of the circulating antigenic variants is necessary for the formulation of a polyvalent vaccine, which would be effective in different malaria-endemic areas [19, 20]. Recently, an alternative approach is the design of a vaccine focusing the immune response toward conserved epitopes that are the target of neutralizing inhibitory antibodies [12]. Understanding mechanisms of sequence variation in *dbpII* alleles and the immunological response to DBPII variant antigens may help in designing vaccines. Therefore, the purpose of this study was to evaluate whether sequence polymorphisms in *dbpII* genes affect the inhibitory function of naturally occurring antibody to eight sequence variants of DBL-TH antigens (DBL-TH1, -TH2, -TH4, -TH5, -TH6, -TH7, -TH8 and -TH9), which may have implications for development of DBPII-based vaccine.

The analysis of genetic diversity of *dbpII* alleles among *P*. *vivax* isolates has been reported from different geographical regions, including Brazil [21], Colombia [22], South Korea [23], Thailand [24], Sri Lanka [25], Iran [26], Myanmar [27] and Papua New Guinea [28, 29]. Among the common polymorphic residues were K371E, D384G, E385K, K386N, N417K, L424I, W437R and I503K [21–30]. Although these polymorphisms do not appear to interfere with receptor recognition, some of them (R308S, D384K and K386N) affect the ability of acquired neutralizing antibodies to inhibit DBP function [8, 10, 13]. In our current study, inhibitory responses against DBL-TH4 and -TH5 haplotypes in high responders showed strain specificity whereas broadly neutralizing inhibition was shown in their response against DBL-TH1, -TH2, -TH6, -TH7, -TH8 and -TH9. This suggests that mutant residues (D384K, K386Q/N, N417K, L424I, W437R, and I503K), which are present in both DBL-TH4 and -TH5, may alter antibody recognition. Additionally, sequence alignment of DBL-TH2 and -TH4 indicated shared common polymorphic residues (L333F, D384G, E385K, K386Q, N417K, L424I, W437R) and only residues, K371E, R390H and I503K were different between DBL-TH2 and -TH4 [14]. I503K has previously been shown to be part of a haplotype that alters sensitivity to inhibition [8]. Therefore, these data suggest that these polymorphic residues contained in the DBL-TH4 haplotype may interfere with strain-transcending immunity of anti-DBPII antibodies.

To understand protective immunity against DBL-TH haplotypes in individuals exposed to *P*. *vivax* infection, the inhibitory function of naturally acquired anti-DBPII antibodies against a panel DBL-TH variants was evaluated in acutely infected *P*. *vivax* patients infected with Sal I or DBL-TH haplotypes. The result demonstrated that patients who were infected with *P*. *vivax* expressing the DBP Sal I allele showed a high prevalence of strain-transcending inhibition towards DBL-TH strains with 50.0% and 40.1% of Sal I patients having broad inhibition against DBL-TH4 and DBL-TH5 haplotypes, respectively, whereas only 13.82% of patients produced antibodies to inhibit homologous Sal I DBP. Additionally, most vivax patients infected with DBL-TH variants had inhibitory antibodies against heterologous DBL-TH4 (33.33%) or DBL-TH5 (27.78%) whereas patients had no inhibitory immune response against heterologous Sal I stain. These data suggested that vivax patients infected with Sal I strain could produce broadly neutralizing antibodies to heterologous *dbpII* Thai variant alleles. However, the patients infected with DBL-TH variants rarely produced broadly neutralizing antibodies against reference Sal I stain. Interestingly, some vivax patients infected with DBL-TH variants had anti-DBPII antibodies to inhibit both homologous and heterologous DBL-TH variants. Together, this study support that DBPII variation is an evasion mechanism responsible for strain-specific immunity and that stable broadly neutralizing antibodies are achieved when antibodies target functionally conserved epitopes.

Strain-transcending immunity of anti-DBPII immune response has been observed in previous studies. Children who had long-term exposure to malaria showed high levels of anti-DBP inhibitory antibody and had strain-transcending protection against *P*. *vivax* infection [5]. This finding is in line with other studies that demonstrated that long-term residents living in vivax malaria endemic areas were able to inhibit erythrocyte binding to two common variants (Sal I and Acre-1) [31]. The cross reactivity of anti-DBPII antibody to heterologous variants (DBPI, DBPV, DBPVI, DBPIX, DBPX) was also demonstrated in Iranian vivax infected individuals [11]. Additionally, a study by *Ntumngia et al* [32] revealed that monoclonal antibodies specific to DBP7.18 variants (accession no. AAL79051.1) recognize conserved epitopes of heterologous *dbpII* alleles including DBPII Thai haplotypes [14]. Therefore, for DBPII vaccine design, it should be possible to produce broadly neutralizing immunity to all the DBPII variants represented in *P*. *vivax* endemic areas. Here, the association of DBPII Thai polymorphisms with broad inhibition of anti-DBPII antibodies was examined in acutely infected *P*. *vivax* patients. Among vivax patients infected with reference Sal I strain, patient no.9 produced broadly neutralizing antibody blocking heterologous DBL-TH and Sal I-erythrocyte binding (Fig 4A) whereas patient no.15 produced allele-specific neutralizing antibodies towards a panel of DBL-TH haplotypes (Fig 4B, *P* < 0.05). Among vivax patients infected with DBPII Thai variants, patient no.25, infected with DBL-TH2, produced neutralizing antibody against homologous DBL-TH2 and heterologous DBL-TH6 and -TH7 (Fig 4C), while patient no.37, infected with DBL-TH4, produced a broader range of neutralizing antibody against heterologous DBL-TH1, -TH5, -TH6, -TH9 and reference Sal I strain but showed no inhibitory capacity against homologous DBL-TH4 variant (Fig 4D). Moreover, vivax patient no.30, infected with DBL-TH12, showed strain-transcending immunity against heterologous DBL-TH2, -TH6, -TH7, -TH8 and -TH9 (Fig 4E). Together, these data suggests that vivax patients infected with DBPII Thai variants are able to produce anti-DBPII neutralizing antibody against some heterologous DBPII Thai variants. These neutralizing antibodies appear to share recognition of conserved B-cell epitopes to overcome the strain specific immunity.

In summary, we have demonstrated that *P*. *vivax* patients in Thai endemic areas are able to produce neutralizing antibodies that can block DBL-TH variants-erythrocyte binding. Moreover, this study further demonstrates that strain-transcending anti-DBPII immunity against heterologous *dbpII* Thai alleles occurs in some vivax infected individuals. Further study is necessary to identify conserved B-epitopes from *P*. *vivax* field isolates that can overcome DBPII variation as one of the biggest challenge of DBPII-based vaccine development.

## Materials and Methods

### Ethics Statement

This study was approved by the Committee on Human Rights Related to Human Experimentation, Mahidol University, and the Ministry of Health, Thailand (MUIRB2012/079.2408). The participant information sheet was written and approved by Committee on Human Rights Related to Human Experimentation, Mahidol University, and the Ministry of Health, Thailand. The informed consent was signed by each participant before the blood sample was collected. The selecting criteria of the patients were as followings: (1) systolic blood pressure not less than 90 mm, (2) body temperature not higher than 40°C, (3) hematocrit not less than 25% and (4) age of 18 or above. Those who did not fit the criteria were excluded. The minority participants were not involved in the study.

### Blood sample preparation

We collected blood samples from acutely infected *P*. *vivax* patients at malaria clinics in Chumphon province, which is in the Southern part of Thailand. The areas are malaria endemic with a high prevalence of *P*. *vivax* infections. One hundred three sera samples were collected in 2011–2012 from *P*. *vivax* patients for screening for anti-DBPII responses and evaluation of strain-transcending inhibition against DBL-TH variants. Forty blood samples were collected in 2014 to study the association of DBL-TH polymorphisms and broadly inhibitory activity of anti-DBPII antibodies in individuals with *P*. *vivax* exposure. Individuals were assigned for DBPII sequence analysis and testing of inhibitory function of antibodies against Thai DBPII haplotypes. Three blood spots were collected on filter paper from each consenting *P*. *vivax* patient for preparation of parasite isolates. Parasite genomic DNA was extracted with a QIAamp DNA mini kit (Qiagen, Valencia, CA, USA). The confirmation of *P*. *vivax* infection was performed by microscopic examination of thin and thick Giemsa-stained blood smears. Blood samples for malaria-naive controls were obtained from 35 healthy volunteers who live in Bangkok and had no history of exposure to *Plasmodium* parasites. Acute *P*. *vivax*-infected volunteers who registered at Malarial Clinics and naïve control subjects were asked for informed consent under the protocol approved by the Ethic Committee on Human Rights Related to Human Experimentation, Mahidol University (MUIRB2012/079.2408).

### Measurement of antibody response to DBPII antigen by ELISA

Serological response against recombinant DBP reference Sal I was quantified by ELISA. Recombinant DBP region II (rDBPII) was expressed as a glutathione S-transferase (GST) fusion protein in E. coli. It was then affinity purified on glutathione and cleaved from GST with thrombin using standard methods [33]. Purified rDBP region II was added to 96-well plates at 2 μg/mL and incubated overnight at 4°C. Wells were incubated with blocking buffer (2% skim milk in PBS) for 2 hr and washed three times with wash buffer. The diluted plasma (n = 103) at 1:200 was added to allow binding to rDBP antigen and incubated for 1 hr at 37°C. Bound rDBP and human plasma were detected with goat anti-human IgG-alkaline phosphatase (1:1000 dilution; KPL, Maryland, USA). The anti-DBPII antibody activity was detected by recording the absorbance (OD) at 405 nm. The overage absorbance and standard deviation were calculated for each plasma sample. A baseline OD was established using plasma from 40 samples of non-malaria exposed Thai individuals and this control value was subtracted from test OD values to standardize the assay. The samples were considered positive when the OD value was greater than or equal to the mean plus 2 standard deviations of negative controls. The antibody reactivity in human plasma was classified into three groups: high responder (OD = 0.41 to 0.69), low responders (OD = 0.20 to 0.40) and non-responders (NR) (OD < 0.20) [10, 13].

### Identification of DBL-TH haplotypes in *P*. *vivax* patients

To identify the DBP region II genotype of the causative agent in acutely infected *P*. *vivax* patients, 40 blood spot samples were taken for DNA isolation and amplification. DBPII genes were PCR amplified as described in detail previously [14]. In brief, PCR cycling conditions for each primer pair were 90 sec initial denaturation at 94°C, followed by 30 cycles of 15 sec denaturation at 94°C, 35 sec annealing at 60°C, and 60 sec extension at 68°C, and a final extension step of 2 min at 68°C. The PCR products were subsequently sequenced using the dideoxynucleotide chain termination method (Applied Biosystems, Foster City, CA). The alignment of complete sequences of PvDBPII genes from 40 isolates were analyzed by CLUSTAL and percent similarity was assessed using BioEdit software.

### COS7 culture and transfection

COS7 cell erythrocyte binding assays were carried out to evaluate the ability of neutralizing antibodies in *P*. *vivax* patient to inhibit binding of DBL-TH variant and reference Sal I haplotypes to human erythrocytes. Expression plasmid constructs were engineered to express DBL-TH or reference Sal I alleles on the surface of transiently transfected COS-7 cells as fusion proteins to the N-terminus of enhanced green fluorescent protein (EGFP) [7]. Recombinant plasmids were transfected into green monkey kidney cells (COS-7, American Type Culture Collection, and Manassas, VA, USA) by the use of Lipofectamine 2000 reagent (Invitrogen Life Technologies, Carlsbad, CA, USA). COS-7 cells were seeded in 24-well culture plates (4.5 × 10^4^ cells/well) in Dulbecco’s Modified Eagle Medium (DMEM, Sigma, USA) with 10% fetal bovine serum (Gibco BRL, Life Technologies, Rockville, MS, USA). Recombinant plasmid DNA (100 ng/well) was mixed with Lipofectamine (2% Lipofectamine 2000/well) in DMEM without serum and then added into transfected wells (100 μl/well) and incubated in a humidified incubator with 5% CO_2_ at 37°C for 42–44 hr. The detection of the C-terminal green fluorescent protein (GFP)-expressing vector was used as positive control for checking transfection efficiency.

### Measurement of the inhibitory efficiency of anti-DBPII antibodies against DBL-TH binding by COS7 cell binding-inhibition assay

The inhibition of anti-DBPII neutralizing antibodies against DBPII-erythrocyte binding was performed as previously reported [7, 34]. Briefly, 42–44 hr after transfection, diluted human plasma from high responders was pre-incubated with the transfected COS-7 cells expressing DBL-TH or reference Sal I haplotypes for 1 hr at 37°C before addition of a 10% suspension of Duffy positive human erythrocytes in each well followed by a 2 hr incubation. Unbound erythrocytes were removed by washing the well with PBS. DBP-erythrocyte binding was quantified by counting rosettes observed over 30 fields of view (magnification, ×200). The binding-inhibition was determined by assessing the number of rosettes in wells of transfected COS7 cells in the presence of plasma relative to rosettes in wells of transfected cells in presence of medium control. The positive and negative inhibition binding controls were 3C9 mouse monoclonal antibodies against DBPII.7.18 haplotype [14] and medium control, respectively. Experiments for each human plasma sample were done in triplicate wells and were repeated two times.

### Statistical analysis

Comparison of anti-DBPII antibody levels between unpaired groups (patients compared to naive controls) was performed using the Mann-Whitney U test. The inhibition activity for each plasma sample was compared between all the DBL-TH alleles and tested for any statistically significant differences in antibody reactivity and inhibitory responses by one-way analysis of variance and multiple comparison analysis by Bonferroni test. *P*-values < 0.05 were considered significant. The statistical analysis was performed and graphs prepared using GraphPad Prism (v. 5; GraphPad Software, San Diego, CA, USA).

## Supporting Information

S1 Table DBPII haplotypes as causative agent of *P*. *vivax* infection in individuals at time of enrollment. Data are presented DBL-TH haplotypes in acutely infected *P*. *vivax* patients (n = 40) Blood spot samples were taken for DNA isolation and amplification. DBPII genes were PCR amplified and PCR products were subsequently sequenced. The alignment of complete sequences of PvDBPII genes from 40 isolates were analyzed by CLUSTAL and percent similarity was assessed using BioEdit software.
(DOCX)

## Abbreviations

DBPII: Duffy binding protein region II
DBL-TH: Duffy binding ligand Thai haplotypes
