# Supplementary material for: Strain-Transcending Inhibitory Antibodies against Homologous and Heterologous Strains of Duffy Binding Protein region II
Source: PLoS One. 2016 May 4;11(5):e0154577. doi: 10.1371/journal.pone.0154577 (PMC4856259; doi:10.1371/journal.pone.0154577)
Supplement: S1 Table — Data are presented DBL-TH haplotypes in acutely infected P. vivax patients (n = 40) Blood spot samples were taken for DNA isolation and amplification. DBPII genes were PCR amplified and PCR products were subsequently sequenced. The alignment of complete sequences of PvDBPII genes from 40 isolates were analyzed by CLUSTAL and percent similarity was assessed using BioEdit software. (DOCX) [file pone.0154577.s001.docx]

**Supplementary data**

**S1 Table:** DBPII haplotypes as causative agent of *P. vivax* infection in individuals at time of enrollment

| Sample ID | DBPII Haplotypes | | | | | | | | | | | | | | | | | | | | | | | | | |
| --- | --- | --- | --- | --- | --- | --- | --- | --- | --- | --- | --- | --- | --- | --- | --- | --- | --- | --- | --- | --- | --- | --- | --- | --- | --- | --- |
|  | Sal I | | TH1 | | TH2 | | TH3 | | TH4 | | TH5 | | TH6 | | TH7 | | TH8 | | TH9 | | TH10 | | TH11 | | TH12 | |
| 1 |  | |  | |  | |  | |  | |  | |  | |  | |  | |  | | + | |  | |  | |
| 2 | + | |  | |  | |  | |  | |  | |  | |  | |  | |  | |  | |  | |  | |
| 3 | + | |  | |  | |  | |  | |  | |  | |  | |  | |  | |  | |  | |  | |
| 4 | + | |  | |  | |  | |  | |  | |  | |  | |  | |  | |  | |  | |  | |
| 5 |  | |  | |  | | + | |  | |  | |  | |  | |  | |  | |  | |  | |  | |
| 6 | + | |  | |  | |  | |  | |  | |  | |  | |  | |  | |  | |  | |  | |
| 7 | + | |  | |  | |  | |  | |  | |  | |  | |  | |  | |  | |  | |  | |
| 8 |  | | + | |  | |  | |  | |  | |  | |  | |  | |  | |  | |  | |  | |
| 9 | + | |  | |  | |  | |  | |  | |  | |  | |  | |  | |  | |  | |  | |
| 10 |  | |  | |  | |  | |  | |  | |  | |  | |  | | + | |  | |  | |  | |
| 11 | + | |  | |  | |  | |  | |  | |  | |  | |  | |  | |  | |  | |  | |
| 12 | + | |  | |  | |  | |  | |  | |  | |  | |  | |  | |  | |  | |  | |
| 13 |  | |  | |  | |  | |  | |  | | + | |  | |  | |  | |  | |  | |  | |
| 14 | + | |  | |  | |  | |  | |  | |  | |  | |  | |  | |  | |  | |  | |
| 15 | + | |  | |  | |  | |  | |  | |  | |  | |  | |  | |  | |  | |  | |
| 16 |  | | + | |  | |  | |  | |  | |  | |  | |  | |  | |  | |  | |  | |
| 17 |  | |  | |  | |  | |  | |  | |  | |  | |  | |  | |  | | + | |  | |
| 18 | + | |  | |  | |  | |  | |  | |  | |  | |  | |  | |  | |  | |  | |
| 19 | + | |  | |  | |  | |  | |  | |  | |  | |  | |  | |  | |  | |  | |
| 20 |  | |  | |  | |  | |  | |  | |  | | + | |  | |  | |  | |  | |  | |
| 21 |  | | + | |  | |  | |  | |  | |  | |  | |  | |  | |  | |  | |  | |
| 22 | + | |  | |  | |  | |  | |  | |  | |  | |  | |  | |  | |  | |  | |
| 23 |  | | + | |  | |  | |  | |  | |  | |  | |  | |  | |  | |  | |  | |
| 24 | + | |  | |  | |  | |  | |  | |  | |  | |  | |  | |  | |  | |  | |
| 25 |  | | + | |  | |  | |  | |  | |  | |  | |  | |  | |  | |  | |  | |
| 26 |  | | + | |  | |  | |  | |  | |  | |  | |  | |  | |  | |  | |  | |
| 27 | + | |  | |  | |  | |  | |  | |  | |  | |  | |  | |  | |  | |  | |
| 28 |  | |  | |  | |  | |  | |  | |  | |  | | + | |  | |  | |  | |  | |
| 29 | + | |  | |  | |  | |  | |  | |  | |  | |  | |  | |  | |  | |  | |
| Sample ID | DBPII Haplotypes | | | | | | | | | | | | | | | | | | | | | | | | |  |
|  | Sal I | TH1 | | TH2 | | TH3 | | TH4 | | TH5 | | TH6 | | TH7 | | TH8 | | TH9 | | TH10 | | TH11 | | TH12 | |  |
| 30 |  |  | |  | |  | |  | |  | |  | |  | |  | |  | |  | |  | | + | |  |
| 31 | + |  | |  | |  | |  | |  | |  | |  | |  | |  | |  | |  | |  | |  |
| 32 | + |  | |  | |  | |  | |  | |  | |  | |  | |  | |  | |  | |  | |  |
| 33 |  | + | |  | |  | |  | |  | |  | |  | |  | |  | |  | |  | |  | |  |
| 34 |  | + | |  | |  | |  | |  | |  | |  | |  | |  | |  | |  | |  | |  |
| 35 | + |  | |  | |  | |  | |  | |  | |  | |  | |  | |  | |  | |  | |  |
| 36 | + |  | |  | |  | |  | |  | |  | |  | |  | |  | |  | |  | |  | |  |
| 37 |  |  | |  | |  | | + | |  | |  | |  | |  | |  | |  | |  | |  | |  |
| 38 | + |  | |  | |  | |  | |  | |  | |  | |  | |  | |  | |  | |  | |  |
| 39 | + |  | |  | |  | |  | |  | |  | |  | |  | |  | |  | |  | |  | |  |
| 40 |  | + | |  | |  | |  | |  | |  | |  | |  | |  | |  | |  | |  | |  |
